# Lavender Oil-Potent Anxiolytic Properties via Modulating Voltage Dependent Calcium Channels

**DOI:** 10.1371/journal.pone.0059998

**Published:** 2013-04-29

**Authors:** Anita M. Schuwald, Michael Nöldner, Thomas Wilmes, Norbert Klugbauer, Kristina Leuner, Walter E. Müller

**Affiliations:** 1 Department of Pharmacology, Biocenter, Goethe University, Frankfurt, Germany; 2 Dr. Willmar Schwabe Pharmaceuticals, Karlsruhe, Germany; 3 Institut für Experimentelle und Klinische Pharmakologie und Toxikologie, Albert-Ludwigs Universität, Freiburg, Germany; 4 Department of Molecular and Clinical Pharmacy, Friedrich-Alexander University, Erlangen, Germany; Alexander Flemming Biomedical Sciences Research Center, Greece

## Abstract

Recent clinical data support the clinical use of oral lavender oil in patients suffering from subsyndromal anxiety. We identified the molecular mechanism of action that will alter the perception of lavender oil as a nonspecific ingredient of aromatherapy to a potent anxiolytic inhibiting voltage dependent calcium channels (VOCCs) as highly selective drug target. In contrast to previous publications where exorbitant high concentrations were used, the effects of lavender oil in behavioral, biochemical, and electrophysiological experiments were investigated in physiological concentrations in the nanomolar range, which correlate to a single dosage of 80 mg/d in humans that was used in clinical trials. We show for the first time that lavender oil bears some similarities with the established anxiolytic pregabalin. Lavender oil inhibits VOCCs in synaptosomes, primary hippocampal neurons and stably overexpressing cell lines in the same range such as pregabalin. Interestingly, Silexan does not primarily bind to P/Q type calcium channels such as pregabalin and does not interact with the binding site of pregabalin, the α2δ subunit of VOCCs. Lavender oil reduces non-selectively the calcium influx through several different types of VOCCs such as the N-type, P/Q-type and T-type VOCCs. In the hippocampus, one brain region important for anxiety disorders, we show that inhibition by lavender oil is mainly mediated via N-type and P/Q-type VOCCs. Taken together, we provide a pharmacological and molecular rationale for the clinical use of the oral application of lavender oil in patients suffering from anxiety.

## Introduction

Lavender oil (LA) is an important part of our today's aromatherapy to promote “well-being” and to reduce distress and “ill-being”. “Well-being” is a psychological construct comprising several domains related to personality including self- acceptance and purpose in life among others [1]. Thus, changes induced by LA might be more directed to improvement of ill-being and distress which show overlap with anxiety and tension at the biological level [2]. When applied by inhalation LA has been associated not only with feelings of pleasantness but also with some improving effects on mood and anxiety [3], [4]. While most if not all of those effects of lavender oil in aromatherapy may be mediated by its pleasant odour there is increasing evidence strongly suggesting a pharmacodynamic effect of LA independent of its odour when applied systemically. I) Anxiolytic properties have been demonstrated for LA in experimental animals following inhalation of very high concentrations but also after i.p. or oral administration [5]–[8]. II) When given in capsules containing 100 or 200 µl LA, anxiolytic properties have been shown in human volunteers following stressful situations [9]. III) Recent clinical trials using Silexan, a standardized LA oil preparation, showed pronounced effects in patients with subsyndromal or subthreshold anxiety disorders as well as in patients with Generalized Anxiety Disorder (GAD) after oral administration. Importantly, Silexan was similarly active compared to the benzodiazepine lorazepam (0,5 mg) during 6 weeks of treatment [10] in patients suffering from GAD. Silexan is a patented active substance produced from Lavandula angustifolia flowers by steam distillation consisting of the main active constituents. linalool (36.8%) and linalyl acetate (34.2%). Silexan (active substance of Lasea®, available as immediate release soft gelatine capules containing 80 mg) has been licensed in Germany for the oral treatment of subsyndromal anxiety and tension in 2009.

Even if several preclinical behavioural pharmacological studies and the new clinical data clearly show the anxiolytic activity of LA and especially of Silexan, the molecular mechanism of action explaining these positive effects was missing. In contrast to previous studies, we used physiological relevant concentrations of Silexan which where found in pharmacokinetic experiments. First, we showed anxiolytic effects of Silexan at these low concentrations in behavioural pharmacological tests such as the elevated plus maze. Second, Silexan showed similar effects compared to the established anxiolytics diazepam and pregabalin. To decipher the molecular mechanism of Silexan, we tested whether Silexan modulates the activity of voltage operated calcium channels (VOCCs) since Silexan did not reveal any affinity to known targets of other anxiolytic drugs (SERT, NET, DAT, MAO-A and the GABA_A_-receptor; data not shown). Under pathological conditions like anxiety or stress disorders, it has been speculated that enhanced Ca^2+^-influx mainly through N and P/Q type VOCCs may increase the release of neurotransmitters such as glutamate and norepinephrine [11], [12] which are involved in the pathogenesis of these diseases. Third, we show for the first time that Silexan unselectively inhibits several VOCCs, such as P/Q-type and N-type VOCCs using a broad set of methods including calcium imaging as well as patch clamp technique. In addition, we were able to demonstrate that Silexan does not bind to the binding site of pregabalin at the P/Q type calcium channels. Pregabalin modulates P/Q type VOCCs after binding at the auxiliary α2δ-1 or -2 subunits and thereby reduces Ca^2+^-influx through these channels [13], [14]. Taken together, we elucidate the anxiolytic mechanism of action of LA and thereby provide a molecular rationale for the clinical use of Silexan.

## Materials and Methods

### Animals

Female 2–3 months old NMRI mice with an average weight of 30 g were used for the preparation of synaptosomes, purified synaptosomal membranes and for the determination of pentobarbital sleep time. Male 2–3 months old NMRI mice were utilized for the elevated plus maze test. For the preparation of primary hippocampal neurons female time-mated Sprague-Dawley rats were used. Animals were purchased from Charles River Laboratories, Sulzfeld, Germany or Janvier SAS, St. Berthevin, France. All animal care and experimental procedures were in concordance with the German law on animal care and handling of animals and the guidance of the European Community Council Directive, and the protocol was approved by the local commission for the Care and Use of Laboratory Animals of the Government by Baden-Württemberg (Regierungspräsidium Karlsruhe, permit numbers 35-9185.82/A-33/04 and 35-9185.82/A-31/04). All animals were housed in plastic cages with water and food *ad libitum* and were maintained on a 12 h light/dark cycle.

### Drugs

Silexan, linalool, linalyl acetate, diazepam and pregabalin were kindly provided by Dr. Willmar Schwabe GmbH & Co. KG, Karlsruhe, Germany. Pentobarbital sodium salt, ω-agatoxin IVA, nifedipine, arachidonylethanolamide, pertussis toxin, geraniol, 1,8-cineole and fura-2-AM were purchased from Sigma-Aldrich, Taufkirchen, Germany. ω-conotoxin GVIA was obtained from Tocris Bioscience, Bristol, UK and dihydrolinalool from TCI Europe, Eschborn, Germany. [^3^H]-gabapentin was obtained from Biotrend, Cologne, Germany.

### Elevated Plus Maze test

Male NMRI mice (8 per group) were treated orally for 3 days with doses between 1 and 30 mg/kg/day Silexan, 0.2% agar suspension (10 ml/kg), diazepam (2.5 mg/kg) or pregabalin (100 mg/kg) as positive controls. Anxiety-related behaviour was tested 1 h after the last treatment in a standard elevated plus maze apparatus (central platform 5×5 cm, open arms 30×5 cm, closed arms 30×5×15 cm), 60 cm above the floor. The number of entries and the time spent in the open arms was recorded during 5 minutes.

### Pentobarbital sleep time

Female NMRI mice were treated orally for 9 days with dosages between 1 and 30 mg/kg Silexan, 0.2% agar suspension (10 ml/kg) or pregabalin (100 mg/kg). Pentobarbital (45 mg/kg) was diluted in physiological saline and administered i.p. to each mouse one hour after last treatment. The sleeping time was defined as the lapse of time required to change from dorsal position to the normal position.

### Isolation and plating of rat P0–P2 hippocampal neurons

Primary hippocampal neurons were prepared from P0–P2 Sprague-Dawley rat pups according to Amaral *et al* using the Worthington Papain Dissociation Kit (Worthington, Lakewood, NJ) [15]. Cells were plated on poly-D-lysine/laminin coated glass cover slips in serum free Neurobasal A media containing L-glutamine (1 mM) and 2% B27 in 6 well plates at a density of 2×10^5^ cells per well. Neurons were grown in 37°C in a humidified incubator containing 5% CO_2_ in air. Cultures were maintained for 14 days before experimental procedures.

### Calcium measurements in primary hippocampal neurons

Intracellular Ca^2+^ measurements were conducted according to literature [16], [17]. After 14 days, hippocampal neurons on cover slips were loaded with 1 µM fura-2-AM for 30 min and then placed on the stage of an inverted Axiovert S100 microscope (Zeiss, Oberkirchen, Germany). Intracellular calcium concentrations were measured by dual excitation (340/380 nm) ratio method.

### Synaptosomal preparations for intracellular calcium measurements

Murine synaptosomes from whole brain without cerebellum were prepared as described previously [18]. Synaptosomal samples were loaded with 5 µM fura-2-AM for 40 min. Fura-2-signals were calibrated according to the method of Grynkiewicz *et al*, using a K_D_ value of 224 nM [19]. For the determination of R_max_ and R_min_ 0,2% sodium dodecyl sulphate and tris(hydroxymethyl)aminomethane (Tris) 30 mM/ethylene glycol bis(2-aminoethyl ether)-N,N,N′N′-tetraacetic acid (EGTA) 6 mM, respectively were added.

### [^3^H]-gabapentin binding studies

Partially purified synaptic membranes were prepared from mouse cortex using sucrose density gradients according to Suman-Chauhan *et al* [20]. Protein content of synaptic membrane suspension was determined by the Lowry protein assay [21]. For [^3^H]-gabapentin binding studies, tissue (0.1 mg protein) was incubated with 20 nM [^3^H]-gabapentin in 10 mM HEPES buffer (pH 7.4 at RT) in the presence of varying concentrations of test compound for 40 min. Afterwards, the samples were filtered through Whatman GF/B filters under vacuum. Filters were washed 3 times with 5 ml ice-cold 100 mM NaCl. Bound radioactivity was determined using liquid scintillation counting. Non-specific binding was defined by 1 mM pregabalin.

### Electrophysiological recordings

For measurement of N- and P/Q-type currents, different transfected cell lines were applied, each expressing only one VOCC subtype. N-type channel recordings were carried out on CHO cells stably expressing Cav2.2, α2δ-1 and β-subunits. The cell line was kindly provided by B. Fakler, Institute of Physiology II, University of Freiburg, Germany. Cells were grown in Minimal Essential Medium (MEM) ALPHA including 10% fetal calf serum, L-glutamine 200 mM, G418 0.7 mg/ml, hygromycin B 0.25 mg/ml and blasticidin 5 µg/ml. For P/Q-type current analysis, HEK 293 cells were transiently transfected with α2δ-1 and β3a subunits, as previously described, whereas the α_1A_ subunit was cloned in a pEGFP-N1 vector [22].

Whole cell calcium currents were recorded using the HEKA-10 patch clamp amplifier (HEKA Electronic, Lambrecht, Germany). For measurement of calcium current inhibition by Silexan, extracellular solution containing vehicle or Silexan was applied by superfusion and currents were recorded at 1 Hz during a 50 ms depolarizing pulse to 20 mV. The extracellular solution contained (in mM): CaCl_2_ 10, tetraethylammonium chloride (TEA-Cl) 125, HEPES 10 and glucose 5. The pH was adjusted to 7.4 using TEA-OH. The intracellular solution contained (in mM): CsCl 110, HEPES 10, EGTA 10, MgCl_2_ 3, Na-adenosine triphosphate (Na-ATP) 3 and guanosine 5-triphosphate tris salt (GTP-Tris) 0.6. The pH was adjusted to 7.2 using CsOH. Patch pipettes were pulled from borosilicate glass tubing (Sutter Instruments, Novato, CA, USA) with input resistance ranged between 1.8 and 4.0 MΩ. Cell capacitance ranged between 12 and 25 pF for N-type cells and 10 and 50 pF for P/Q-type cells. Series resistance was typically between 2.5 and 8 MΩ and was compensated up to 70%. Data were recorded with HEKA pulse 8.5 software package and analysed off-line. The holding potential in experiments with N- or P/Q-type cells was −80 mV.

### Statistical analysis

Results were expressed as mean ± SEM. Statistical analyses were performed with two-sided t-tests. Stars represent p-values: ***p<0.001, **p<0.01, *p<0.05.

## Results

### Silexan improves anxiolytic behaviour in the elevated plus maze test

**Figure 1:**
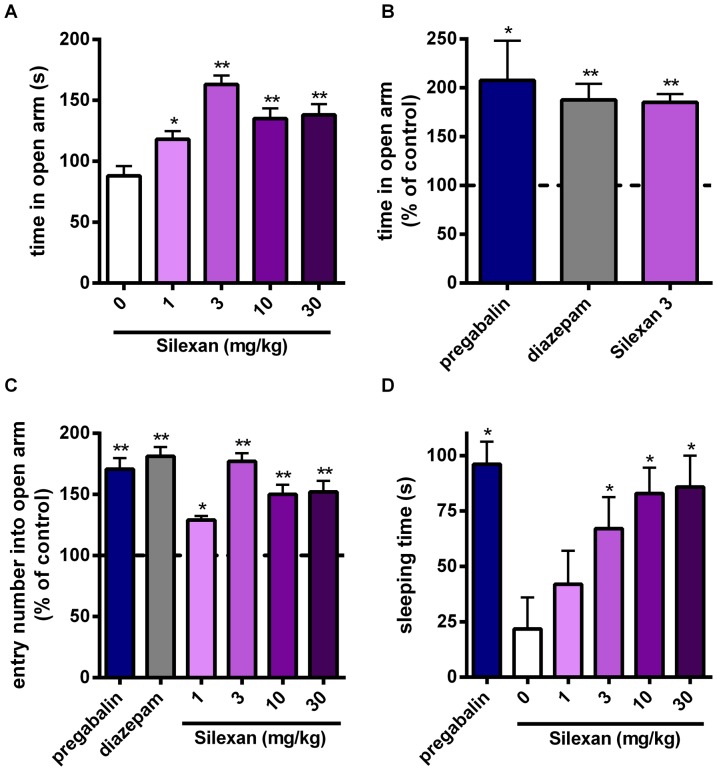
Silexan reduces anxiety-related behaviour in the elevated plus maze and increases pentobarbital-induced sleeping time. (A–C) Mice were treated orally with diazepam (2.5 mg/kg BW), pregabalin (100 mg/kg BW) or Silexan (1–30 mg/kg BW) for three days. Diazepam, pregabalin and Silexan increased the time spent in the open arm (A, B) and entry number (C). (D) Mice were treated orally with pregabalin (100 mg/kg BW) or Silexan (1–30 mg/kg BW) for nine days. Sleeping time was determined after i.p. application of pentobarbital (45 mg/kg BW). All data presented are mean values ± SEM using 8 mice per treatment group (unpaired t-test).

Several groups previously showed anxiolytic effects of LA preparations given by inhalation not standardized concerning their composition [6], [7]. None of these studies dealt with anxiolytic effects of oral administered LA in dosages which are in accordance with the dosage of 80 mg/d used in humans. Therefore, we tested the anxiolytic activity of Silexan in the elevated plus maze test, a well established anxiety model. Mice were treated orally for 3 days with Silexan, the benzodiazepine diazepam or the gabapentinoid pregabalin as active controls. As expected, diazepam and pregabalin both increased the time spent in the open arm (Fig. 1B and C) as well as the number of entries into the open arm. Already at a concentration of 1 mg/kg BW Silexan showed a significant elevation of the number of entries into the open arm (Fig. 1A). This anxiolytic effect is dose-dependent and already maximal at a concentration of 3 mg/kg BW. Similar to diazepam and pregabalin, Silexan also elevated the pentobarbital sleeping time (Fig. 1D) at similar concentrations (3–30 mg/kg) in accordance with Linck *et al* [8]. As Silexan does not show sedation in humans, this finding rather suggests sleep promoting than sedative properties [9], [10], [23]–[26].

### Similar effects of Silexan and pregabalin on VOCCs in murine synaptosomes

**Figure 2:**
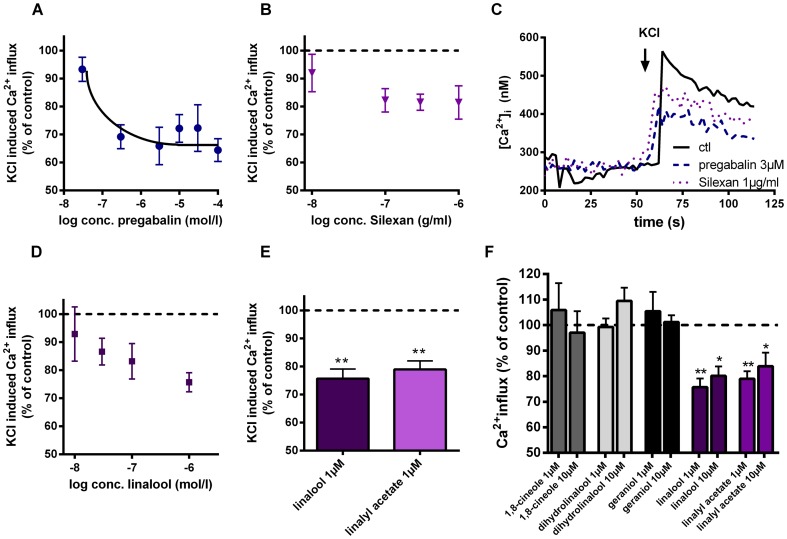
Silexan dose-dependently inhibits VOCCs in murine synaptosomes. Dose-dependent effects of pregabalin (A), Silexan (B) and linalool (D) on KCl-induced Ca^2+^-influx. Murine synaptosomes were preincubated (10 min) with different concentrations of pregabalin (A, 0.03–100 µM), Silexan (B, 0.01–10 µg/ml) or linalool (D, 0.01–1 µM) and afterwards stimulated with KCl (80 mM). (C) Representative Ca^2+^-transients in murine synaptosomes after KCl-induced activation of VOCCs in the presence and absence of pregabalin (3 µM) and Silexan (1 µg/ml). (E) Effect of the preincubation (10 min) with linalool (1 µM) and linalyl acetate (1 µM) on KCl-induced Ca^2+^-influx in murine synaptosomes. (F) Several other monterpenes have no effect on KCl-induced Ca^2+^-influx in murine synaptosomes under similar conditions. All data presented are mean values ± SEM (n = 10–12), paired t-test.

Silexan showed no affinity to known targets of other anxiolytic drugs such as SERT, NET, DAT, MAO-A and the GABA_A_-receptor (data not shown). Therefore, we investigated if Silexan shares its mode of action with the gabapentinoid pregabalin which targets the α2δ-1 and -2 subunits of VOCCs. We examined, if Silexan and pregabalin inhibit Ca^2+^-influx in murine synaptosomes (Fig. 2A–C). VOCCs were activated by KCl. Under these conditions pregabalin inhibited Ca^2+^-influx substantially (Fig. 2A, 2C). Maximal effects were already seen at a concentration of 3 µM reducing the Ca^2+^-influx to 65.86±6.71%. Increasing pregabalin concentrations did not show any additional effect. Silexan dose-dependently reduced KCl-induced Ca^2+^-increase in a biphasic mode, showing an inhibition of about 20% at concentrations between 0.1 and 1 µg/ml(Fig. 2B, 2C). Contrary to pregabalin, Silexan further decreased Ca^2+^-elevation at higher concentrations, exceeding pharmacologically relevant plasma levels in man and animal by several orders of magnitude (data not shown). The major ingredients of Silexan are linalool and linalyl acetate which each make up to about 35% of the product. However, linalool and linalyl acetate are similarly active when applied directly to the synaptosomes (Fig. 2E). Due to the fact that linalyl acetate is completely metabolized to linalool, we estimated an IC_50_ value for linalool of about 37 nM (Fig. 2D).

One possible explanation for this effect could be a nonspecific modulation of channels properties by alterations of membrane fluidity. However, using the fluorescence probe 1,6-diphenyl-1,3,5-hexatriene and mouse brain membranes according to Kirsch et al. [27], no effect of Silexan on membrane fluidity was seem up to concentrations of 100 µg/ml.

To further investigate if the Silexan and linalool mediated effects on VOCCs are selective, we additionally investigated several terpenes with closely related chemical structures such as geraniol, cineol and dihydrolinalool. Importantly, none of the other terpenes inhibited KCl induced calcium influx (Fig. 2F) supporting the idea that this effect is specific for linalool and lavender oil and is not a common phenomenon of lipophilic monoterpenes.

### Silexan also inhibits VOCCs in primary hippocampal neurons

**Figure 3:**
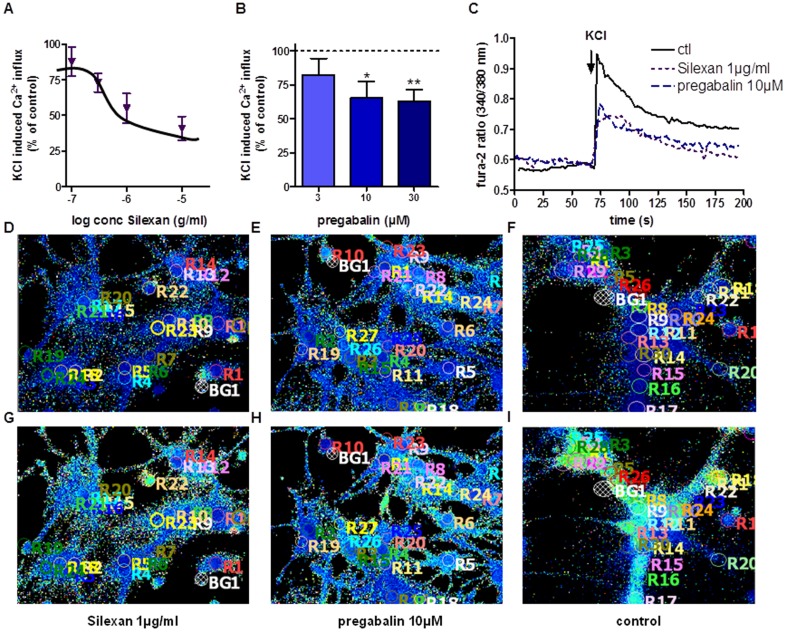
Silexan and pregabalin show similar inhibitory effects on VOCCs in primary hippocampal neurons. Concentration-dependent effect of Silexan (A, 0.01–10 µM) and pregabalin (3–30 µM) after 10 min preincubation on KCl (60 mM) induced Ca^2+^-influx in primary hippocampal neurons. (C) shows representative traces of intracellular Ca^2+^ concentration in primary hippocampal neurons stimulated with KCl (60 mM) preincubated with Silexan (1 µg/ml), pregabalin (10 µM) compared to ctl. (D)–(F) show representative fields of fura-2 loaded hippocampal neurons preincubated with Silexan 1 µg/ml (D), pregabalin 10 µM (E) or control (F) under basal conditions. (G)–(I) show the same samples after stimulation with KCl (60 mM). All data presented are means ± SEM (n = 10–14, paired t-test).

To further characterize the effects of Silexan on VOCCs, we decided to study KCl dependent Ca^2+^-influx in primary hippocampal neurons because of the important role of the hippocampus for regulating emotions and anxiety. Similar to the effects obtained in synaptosomes, Silexan inhibited significantly and dose-dependently KCl induced Ca^2+^-increase (Fig. 3A, C, D, G) in concentrations between 0.1 and 30 µg/ml. In this model, pregabalin decreased Ca^2+^ currents to 62.84±8.64% of ctl (30 µM; Fig.3B, C, E, H).

### Silexan does not bind to the gabapentin binding site at α_2_δ-subunits of P/Q-type VOCCs

**Figure 4:**
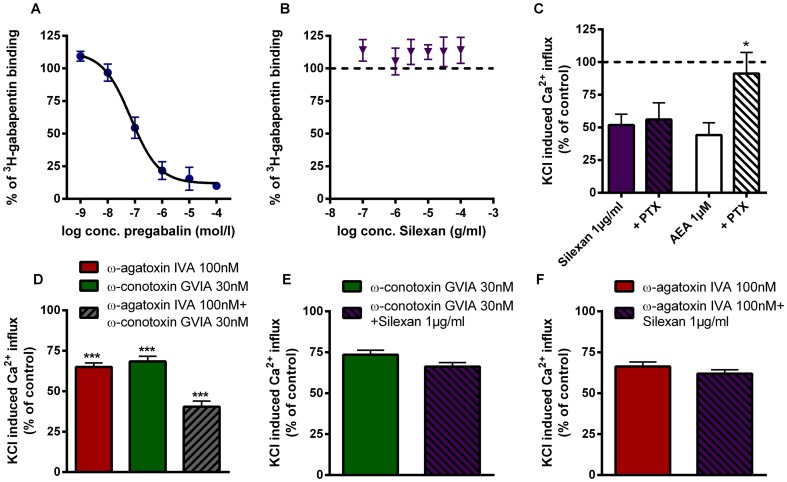
Silexan does neither share the binding site of pregabalin, nor inhibits VOCCs via G_i_-coupled receptors. (A) Displacement studies conducted with [^3^H]-gabapentin in partially purified synaptic membranes. Synaptic membranes were incubated with [^3^H]-gabapentin in presence of pregabalin (A, 0.001–100 µM) or Silexan (B, 0.1–100 µM; n = 3). (C) Primary hippocampal neurons were incubated in the presence or absence of PTX (200 ng/ml) for 18 h. Afterwards cells were treated with Silexan (1 µg/ml) or AEA (1 µM) for 10 min and then stimulated with KCl (60 mM; n = 11–12). (D) Additive inhibitory effects of P/Q-type and N-type channel blockers on KCl-induced Ca^2+^-influx in murine synaptosomes. Synaptosomes were preincubated with ω-agatoxin IVA (100 nM), ω-conotoxin GVIA (30 nM) or a combination of both inhibitors for 10 min. Afterwards, they were stimulated with KCl (80 mM). Silexan (1 µg/ml) causes no significant additive effects when combined with the N-type VOCCs inhibitor ω-conotoxin GVIA (30 nM, E), or the P/Q-type inhibitor ω-agatoxin IVA (100 nM, F; n = 9–12). All data presented are mean values ± SEM (paired t-test).

The specific effects of pregabalin and the structurally related drug gabapentin on presynaptic VOCCs of the P/Q-type have been associated with the specific binding to the modulating and partially extracellularly located subunit α2δ-1 and -2, which can be labelled by [^3^H]-gabapentin binding [13], [28]. In agreement with the literature pregabalin inhibited specific [^3^H]-gabapentin binding with an IC_50_ value of about 70 nM (Fig. 4A) [29]. In contrast, no effect on [^3^H]-gabapentin binding was observed for Silexan (Fig. 4B). Thus, even if both drugs seem to interact with VOCCs, they probably do not share the same binding site at VOCCs.

### G_i_ coupled G-protein receptors are not involved in VOCCs inhibition by Silexan

Modulation of VOCCs could either occur via direct action on the VOCC protein complex or indirect via activation of upstream G_i_ coupled receptors, e.g. cannabinoid receptors [30]. We measured KCl-induced Ca^2+^-elevation in primary hippocampal neurons treated with Silexan in the presence or absence of pertussis toxin (PTX). PTX specifically and irreversibly inactivates G_i_ protein coupled receptors via catalysis of G_αi_ subunit ADP-ribosylation [31]. Silexan reduced significantly depolarisation induced Ca^2+^- influx independent of PTX treatment (Fig. 4C). Arachidonylethanolamide was used as positive control. The endogenous ligand for the CB_1_ cannabinoid receptor decreased KCl-induced Ca^2+^-influx to 44.26±9.37% [32]. Due to G_i_ protein involvement, the inhibitory effect was almost completely abolished after PTX treatment (91.28±16.22%). Taken together, Silexan does not interact with G_i_ coupled G-protein coupled receptors.

### Silexan inhibits different classes of VOCCs

To elucidate if Silexan preferentially inhibits P/Q type VOCCs such as pregabalin, we deternined the effects of Silexan in the presence of the P/Q type inhibitor ω-agatoxin IVA and the N-type inhibitor ω-conotoxin GVIA because these two channel types mainly promote the KCl mediated Ca2+-increase in primary hippocampal and in synaptosomes (please also see Supplementary Figure S1).

Both toxins used in this study target ligand-binding pockets at the α_1_ subunit and have been reported to act additively [33], [34]. This was confirmed in our experiments. After single toxin incubation, ω-agatoxin IVA reduced KCl-induced Ca^2+^-elevation to 65.02±8.88%, while ω-conotoxin GVIA diminished Ca^2+^-currents to 68.44±3.16%. Joint application of the two toxins lowered KCl-evoked increase in intrasynaptosomal Ca^2+^-concentrations to 40.43±3.55% (Fig. 4D). To test if Silexan acts specifically at a single VOCC subtype, we performed combined application measurements with ω-conotoxin GVIA (30 nM; Fig. 4E) or ω-agatoxin IVA (100 nM; Fig. 4F). When both toxins were applied together with Silexan, no relevant additive effects were observed. This observation strongly argues against a specific effect of Silexan at one of the individual VOCCs.

**Figure 5:**
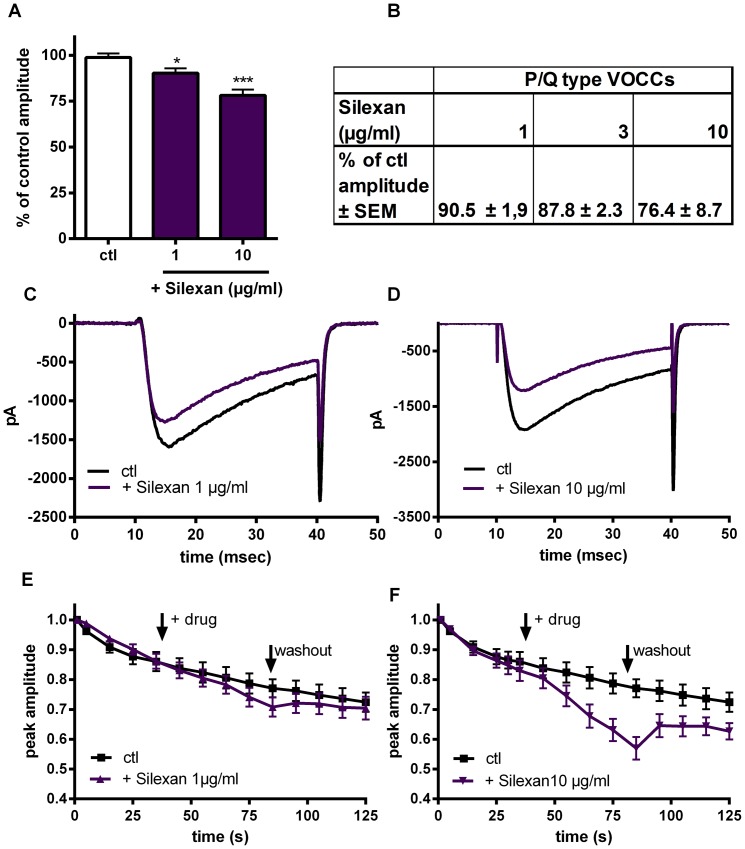
Silexan inhibits N- and P/Q-type VOCCs in whole cell patch-clamp experiments. (A) Whole cell recordings of N-type VOCCs were conducted in CHO cells stably expressing N-type Ca^2+^ channels. Silexan (1 or 10 µg/ml) was applied by superfusion for 50 s. The cells were stimulated with a depolarizing pulse to 20 mV at 1 Hz and the amplitude was recorded (n = 9–14). (C, D) Representative currents of whole cell patch clamp recordings with Silexan 1 and 10 µg/ml in N-type cells when stimulated with a depolarizing pulse to 20 mV before (ctl) and at the end of Silexan application (+ Silexan). (B) Effect of preincubation of Silexan (1–10 µM) on depolarizing pulses to 20 mV in P/Q-type cells (n = 4–5). (E and F) Time course of the N-type Ca2+ channel peak current amplitude for 125 seconds by depolarizing pulses to +20 mV at a pulse frequency of 1 Hz. Inhibition of the N-type Ca2+ channel peak current amplitude by Silexan 1 µg/ml (E) or 10 µg/ml (F) compared to DMSO. The arrows indicate the time points of the Silexan application and the washout. A total number of 10 (DMSO), 14 (Silexan 1 µg/ml) and 9 cells (Silexan 10 µg/ml) were averaged. All data presented are mean values ± SEM (unpaired t-test).

This last observation prompted us to further investigate the effects of Silexan on the properties of VOCCs using the patch-clamp technique. In CHO cells stably expressing N-type VOCC, Silexan displayed a concentration-dependent inhibition of Ca^2+^ currents (Fig. 5A/C/D). Silexan 1 µg/ml already reduced Ca^2+^ currents significantly by about 10%. At the higher concentration of 10 µg/ml Ca^2+^ currents were additionally inhibited to about 80%. The currents recover after washing out Silexan 1 µg/ml and 10 µg/ml (Fig. 5 E,F). Silexan also affected other VOCCs. This could be demonstrated in transiently transfected HEK 293 cells, expressing the P/Q-type channel (Fig. 5B). Silexan caused a reduction of Ca^2+^ currents in P/Q-type expressing cells to a similar extent and in a similar dose-dependent fashion. Electrophysiological recordings in HEK 293 cells stably expressing the T-type VOCC revealed that Silexan also inhibits low voltage activated currents at this channel (data not shown). Taken together, our findings suggest that Silexan acts non-specifically at different classes of VOCC.

## Discussion

In the past, several mechanisms for lavender oil's beneficial effects on anxiety and mood were postulated, including interactions with NMDA or GABA_A_ receptors, voltage dependent sodium channels, or glutamatergic and cholinergic neurotransmission [35]–[39]. Most of these effects were observed in the millimolar or high micromolar concentration range.

Here we show three novel findings. First, Silexan shows anxiolytic effects in the elevated plus maze test at low oral doses (1–10 mg/kg BW) corresponding to dosages given in humans (80 mg/d). Second, our studies revealed an inhibition of VOCCs by Silexan at nanomolar concentrations. Third, pregabalin and Silexan do not share the molecular target, the α2δ subunit of P/Q type channels [13], [40]. In addition, Silexan did not inhibit Ca^2+^-increase via VOCCs as a consequence of previous activation of G_i_ coupled receptors. Silexan unselectively modulates with similar potency several VOCCs such as the N-type and the P/Q type VOCCs. This parallels to some extent pregabalin, which acts on the VOCC channel complex via the α2δ subunit but mainly inhibits P/Q type calcium channels. The precise mechanisms of the channel modulation by Silexan are not yet known but may be either direct by binding and inhibiting the channel complex or more indirect by influencing the properties or surface expression of VOCC subunits.

Taken together, we shed light into the molecular mechanism of action of LA in anxiety and thereby might help to further establish the clinical use of Silexan in patients suffering from anxiety.

## Supporting Information

Figure S1 **Similar expression pattern of VOCCs in murine synaptosomes and primary hippocampal neurons.** μ Murine synaptosomes (A–C) and primary hippocampal neurons (D–F) were preincubated with the selective P/Q type blocker ω-agatoxin IVA (A/D), the selective N type blocker ω-conotoxin GVIA (B/E) and the L type blockers verapamil and nifedipine (C/F) and afterwards stimulated with KCl. All data represent mean ± SEM (n = 8–12; paired t-test). (G)–(J) Representative confocal images of primary hippocampal neurons stained with a specific primary antibody against P/Q-type VOCCs (G), N-type VOCCs (H), and L-type VOCCs (I,J).
(TIF)

Data S1 **Supplementary Data.**
(DOCX)
