# Supplementary material for: Lavender Oil-Potent Anxiolytic Properties via Modulating Voltage Dependent Calcium Channels
Source: PLoS One. 2013 Apr 29;8(4):e59998. doi: 10.1371/journal.pone.0059998 (PMC3639265; doi:10.1371/journal.pone.0059998)
Supplement: Data S1 — Supplementary Data. (DOCX) [file pone.0059998.s002.docx]

**Supplementary data**

**Methods**

*Immunostaining and confocal microscopy.* Primary hippocampal neurons were prepared as described above and plated on poly-D-lysine/ laminin coated glass cover slips in 24 well plates at a density of 5X10^4^ cells per well. Cells were maintained in culture for 14 days, then fixed in 4 % paraformaldehyde for 20 min, blocked in 3% bovine serum albumin solution and permeabilized with phosphate-buffered saline (PBS) containing 0.2% Triton-X-100. Primary hippocampal neurons were then incubated with rabbit anti-CACNA1A, anti-CACNA1B, anti-CACNA1C or anti-CACNA1D antibodies (1:200 dilution in PBS; EMD Millipore, Billerica/ MA, USA) overnight at 4°C. The samples were then washed 3 times with PBS and incubated with Alexa Fluor 488-conjugated anti-rabbit secondary antibody (1:250 dilution in PBS, Invitrogen, Karlsruhe, Germany) for 1 hour at room temperature. Primary hippocampal neurons were embedded in Mowiol and analyzed with the confocal laser scanning microscope TCS SP5 (Leica, Wetzlar, Germany).

**Results**

*Characterisation of VOCCs in murine synaptosomes and primary hippocampal neurons.* Considering that Silexan does not share the binding site of pregabalin at P/Q-type VOCCs and also does not interact with G_i_ protein coupled receptors, we compared its effects with well-known inhibitors of VOCCs in the two systems used, murine synaptosomes and primary hippocampal neurons [40]. The inhibitory potencies of toxins which selectively target one channel subtype such as the P/Q-type channel inhibitor ω-agatoxin IVA, the N-type channel blocker ω-conotoxin GVIA, or the L-type inhibitors nifedipine and verapamil were investigated.

KCl-induced Ca^2+^-influx into murine synaptosomes could be reduced dose-dependently up to 50.1 ± 3.2% by the P/Q-type inhibitor ω-agatoxin IVA (1µM; Fig. 1S, A). The N-type blocker ω-conotoxin GVIA (Fig. 1S, B) displayed an U-shaped inhibition on KCl-induced Ca^2+^-elevation as previously described [40]. The maximal reduction could be observed at a concentration of 30nM (61.7 ± 3.2%). In agreement with the minor role of L-type Ca^2+^ channels for this system, the L-type inhibitor verapamil (1µM; Fig.1S, C) only showed a decrease of KCl-induced Ca^2+^ influx to 86.7 ± 4.6% [41,42].

In primary hippocampal neurons, KCl induced Ca^2+^-influx could be diminished by ω-agatoxin IVA (200nM; Fig. 1S, D, G) to 54.7 ± 14.4%, implying a high P/Q-type channel involvement. The N-type inhibitor ω- conotoxin GVIA displayed a reduction of depolarization induced Ca^2+^-influx to 69.3 ± 9.1% (30nM; Fig. 1S, E, H), whereas L-type blockade by nifedipine 10µM could only achieve an inhibition of about 27% (Fig. 1S, F, I, J). Similar to our observations in murine synaptosomes, L-type VOCCs only contribute to a small part of KCl-induced Ca^2+^increase in this model.

Taken together, N- and P/Q-type channels seem to play the major role in depolarization evoked increase in murine synaptosomes and primary hippocampal neurons.

40. Field MJ, Cox PJ, Stott E, Melrose H, Offord J, et al. (2006) Identification of the alpha(2)-delta-1 subunit of voltage-dependent calcium channels as a molecular target for pain mediating the analgesic actions of pregabalin. Proc Natl Acad Sci U S A 103: 17537-17542.

41. Werth JL, Hirning LD, and Thayer SA (1991) Omega-Conotoxin Exerts Functionally Distinct Low and High-Affinity Effects in the Neuronal Cell-Line Ng108-15. Mol Pharmacol 40: 742-749.

42. Meder W, Fink K, and Gothert M (1997) Involvement of different calcium channels in K+- and veratridine-induced increases of cytosolic calcium concentration in rat cerebral cortical synaptosomes. Naunyn-Schmiedebergs Arch Pharmacol 356: 797-805.
